# Biventricular myocardial strain analysis in patients with arrhythmogenic right ventricular cardiomyopathy (ARVC) using cardiovascular magnetic resonance feature tracking

**DOI:** 10.1186/s12968-014-0075-z

**Published:** 2014-10-07

**Authors:** Philipp Heermann, Dennis M Hedderich, Matthias Paul, Christoph Schülke, Jan Robert Kroeger, Bettina Baeßler, Thomas Wichter, David Maintz, Johannes Waltenberger, Walter Heindel, Alexander C Bunck

**Affiliations:** Department of Clinical Radiology, University Hospital of Muenster, Albert-Schweitzer-Campus 1, 48149 Münster, Germany; Department of Radiology, University Hospital of Cologne, Cologne, Germany; Department of Cardiology, University Hospital of Muenster, Münster, Germany; Department of Cardiology, Niels-Stensen-Kliniken, Marienhospital Osnabrueck, Osnabrueck, Germany

**Keywords:** Feature tracking, Myocardial strain analysis, Arrhythmogenic right ventricular cardiomyopathy (ARVC), Cardiovascular magnetic resonance

## Abstract

**Background:**

Fibrofatty degeneration of myocardium in ARVC is associated with wall motion abnormalities. The aim of this study was to examine whether Cardiovascular Magnetic Resonance (CMR) based strain analysis using feature tracking (FT) can serve as a quantifiable measure to confirm global and regional ventricular dysfunction in ARVC patients and support the early detection of ARVC.

**Methods:**

We enrolled 20 patients with ARVC, 30 with borderline ARVC and 22 subjects with a positive family history but no clinical signs of a manifest ARVC. 10 healthy volunteers (HV) served as controls. 15 ARVC patients received genotyping for Plakophilin-2 mutation (PKP-2), of which 7 were found to be positive. Cine MR datasets of all subjects were assessed for myocardial strain using FT (TomTec Diogenes Software). Global strain and strain rate in radial, circumferential and longitudinal mode were assessed for the right and left ventricle. In addition strain analysis at a segmental level was performed for the right ventricular free wall.

**Results:**

RV global longitudinal strain rates in ARVC (−0.68 ± 0.36 sec^−1^) and borderline ARVC (−0.85 ± 0.36 sec^−1^) were significantly reduced in comparison with HV (−1.38 ± 0.52 sec^−1^, p ≤ 0.05). Furthermore, in ARVC patients RV global circumferential strain and strain rates at the basal level were significantly reduced compared with HV (strain: −5.1 ± 2.7 vs. -9.2 ± 3.6%; strain rate: −0.31 ± 0.13 sec^−1^ vs. -0.61 ± 0.21 sec^−1^). Even for patients with ARVC or borderline ARVC and normal RV ejection fraction (n=30) global longitudinal strain rate proved to be significantly reduced compared with HV (−0.9 ± 0.3 vs. -1.4 ± 0.5 sec^−1^; p < 0.005). In ARVC patients with PKP-2 mutation there was a clear trend towards a more pronounced impairment in RV global longitudinal strain rate. On ROC analysis RV global longitudinal strain rate and circumferential strain rate at the basal level proved to be the best discriminators between ARVC patients and HV (AUC: 0.9 and 0.92, respectively).

**Conclusion:**

CMR based strain analysis using FT is an objective and useful measure for quantification of wall motion abnormalities in ARVC. It allows differentiation between manifest or borderline ARVC and HV, even if ejection fraction is still normal.

## Background

Arrhythmogenic right ventricular cardiomyopathy (ARVC) is a myocardial disorder characterized by a progressive fibrofatty degeneration preferentially affecting the right ventricular (RV) myocardium [1]. Typical associated manifestations comprise potentially fatal ventricular arrhythmias, electrocardiographic repolarisation and depolarisation changes, structural abnormalities as well as biventricular regional and global dysfunction.

With the fibrofatty degeneration characteristically beginning in the subepicardial myocardial layer and often being focal in distribution, myocardial biopsy usually has only a low yield in detecting the ARVC defining histopathological changes, especially in early disease stages [2]. Instead, the diagnosis of ARVC relies on the presence of a set of major and minor criteria that were defined by an International Task Force Group in 1994 and encompass structural, functional, electrocardiographic and histopathological as well as genetic factors [3]. Herein, imaging plays a pivotal role for the detection of structural and functional abnormalities. Due to its excellent capability in differentiating fatty from myocardial tissue, its high sensitivity for the detection of scar tissue and its good reproducibility in the quantification of right ventricular dimensions, Cardiovascular Magnetic Resonance (CMR) is nowadays commonly used in the diagnostic workup of patients suspected of suffering from ARVC [4].

In 2010 the task force criteria (TFC) were revised with the aim of improving diagnostic accuracy [5]. While the original criteria were prone to significant inter-observer variability due to the use of a qualitative severity grading for ventricular dilatation and systolic dysfunction, the revised criteria define specific cut-off values for end-diastolic volume and ejection fraction. Despite the inclusion of quantitative metrics, the modified TFC still require features that rely on a subjective image interpretation. Accordingly, ventricular dilatation or dysfunction needs to be accompanied by the visual identification of regional wall motion abnormalities in order to qualify as minor or major disease defining criteria. The adherence to subjective features has been viewed as a persistent weakness of the current TFC [6].

The complex right ventricular contraction pattern as well as the occurrence of minor contraction abnormalities, even in healthy subjects, significantly hamper a reproducible differentiation of abnormal from normal contraction patterns and complicate the detection of wall motion abnormalities particularly in the early disease stage [7]. Strain imaging, which quantifies myocardial deformation, has been proposed as a less observer-dependent quantitative estimate for global and regional myocardial contraction [8,9]. Several studies have demonstrated its superiority over conventional functional measures in the detection of early forms of myocardial dysfunction [10-15].

Until recently, strain analysis in clinical routine has been the mainstay of echocardiography [8]. While CMR based techniques for strain analysis like myocardial tagging or tissue phase contrast imaging have been available for some time, these techniques are technically demanding and have not reached clinical routine [16,17].

With the advent of new computational image analysis tools, it is now possible to derive strain values from conventional cine CMR in a retrospective manner by using a feature-tracking algorithm [18]. There are a number of recent studies that suggest that this new technique may play a role in early detection and objective quantification of contraction abnormalities in a variety of cardiac pathologies [19-25].

The aim of the current study was to examine whether CMR based strain analysis using feature-tracking (FT) can serve as a quantifiable measure to confirm global and regional ventricular dysfunction in ARVC patients and support the detection of even early forms of ARVC, when global volumetric parameters are still normal.

## Methods

### Study population

After obtaining approval from our local ethics committee, a retrospective study was performed. We enrolled 72 patients that had been consecutively referred to our department for CMR due to a suspected or clinically confirmed diagnosis of ARVC (n=50) or for screening purposes due to a positive family history for ARVC but with no further signs of manifest disease (n=22). Prior to CMR imaging, written informed consent was obtained from all subjects. The final diagnosis of ARVC was established according to the Task Force Criteria with all subjects having been assessed for the presence of minor and major criteria. Accordingly, the diagnosis was made when two major, one major plus two minor or four minor criteria from different categories were present. A diagnosis of ARVC was made in 20 out of the 50 subjects suspected of suffering from ARVC. The remaining 30 subjects fulfilled only a fraction of the criteria and were classified as borderline ARVC.

CMR images from 10 healthy volunteers (HV) served as a control group. With ARVC being a genetically determined heart muscle disorder, a number of mutations in genes encoding for structural proteins of myocardial cells have been identified to be associated with ARVC. In 15 out of the 20 ARVC patients results from genotyping for the presence of a Plakophilin-2 mutation (PKP-2), a proteinaceous component of myocardial desmosomes, were available.

### CMR

All CMR scans were performed on a 1.5-Tesla system (Achieva, Philips, Best, The Netherlands). A balanced steady-state free precession (b-SSFP) sequence in breath-hold technique and with retrospective ECG-triggering was acquired for functional analysis and subsequent feature tracking analysis. The imaging parameters were set as follows: echo time (TE) and repetition time (TR) were set to shortest resulting in an average TR of around 4 ms and a TE of 2 ms slightly varying with slice orientation. 25 phases per cardiac cycle were acquired. The acquired in-plane resolution depended on the field of view (set according to the patient’s physique) with a mean reconstructed pixel size of 1.7 ± 0.2 mm × 1.5 ± 0.2 mm. The slice thickness was 8 mm for both axial and short axis planes. The sequences were exported in DICOM-Format without special adjustments.

In all patients, the CMR protocol included a stack of cine images in axial orientation covering the entire right ventricle and in short axis orientation covering the entire left ventricle to perform volumetry of the right and left ventricle, respectively.

In addition to quantitative volumetric analysis, cine images were assessed visually for the presence of right ventricular wall motion abnormalities by two experienced observers (ACB and DM) in consensus. Severity of wall motion abnormalities was rated depending on the detection of hypokinetic, akinetic, dyskinetic or aneurysmal segments. For quantitative and qualitative analysis, all readers were blinded to clinical data. Qualitative image analysis and volumetry were performed on a standard post-processing platform (View Forum, R5.1 V1 L2, Philips, Best, The Netherlands).

### Myocardial strain analysis using CMR feature tracking

**Figure 1:**
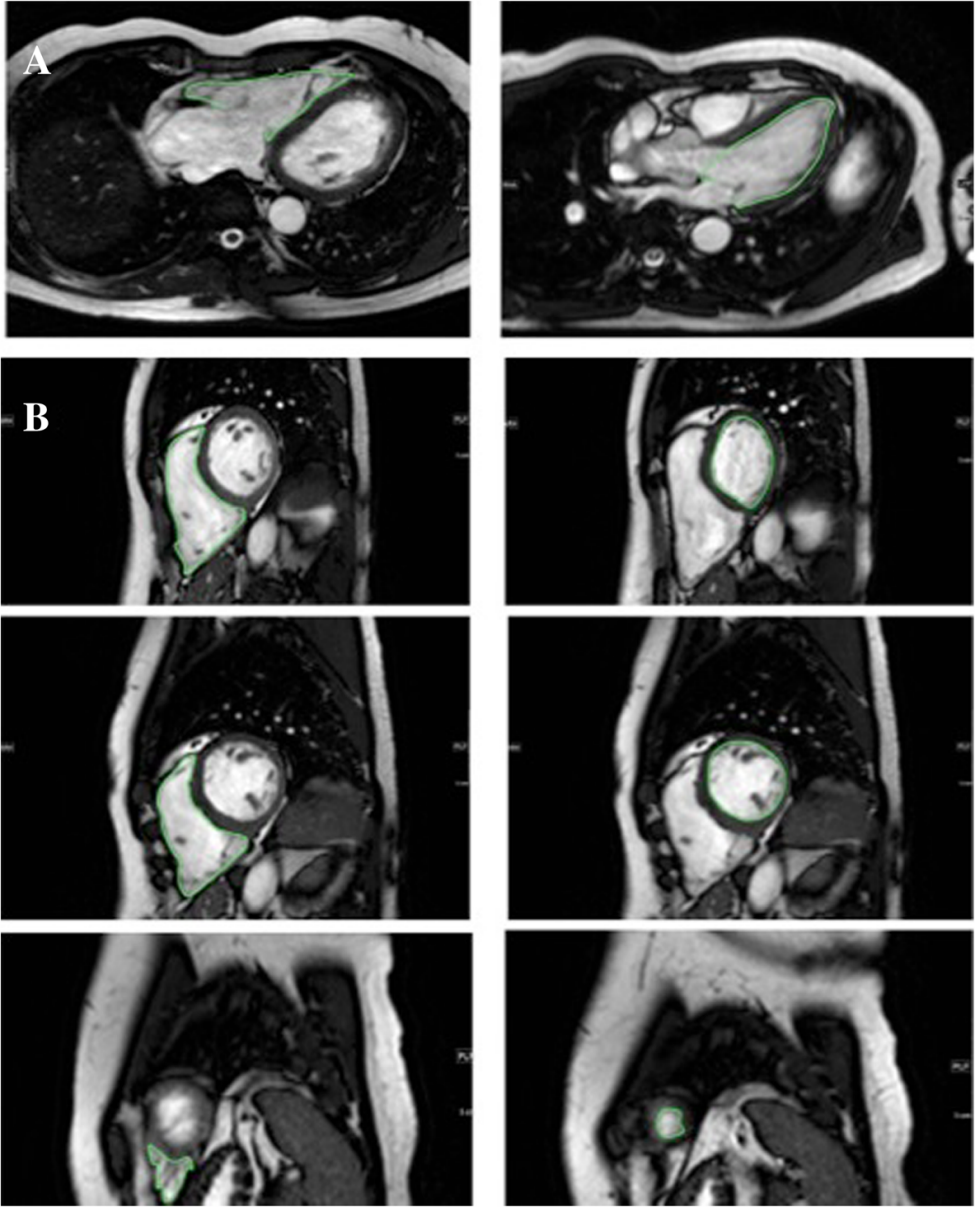
**Tracked right and left ventricular endocardial contours in axial (A) and short axis orientation (B) at the apical, mid-ventricular and basal level.**

The CMR FT analysis was performed on the acquired b-SSFP cine images using a dedicated software (Diogenes MRI, Tomtec, Germany). The details of the feature-tracking algorithm have been published previously [18]. The axial view at the midportion of the corresponding atrioventricular valve was used to derive right ventricular (RV) and left ventricular (LV) longitudinal strain values (Figure 1A). In addition, a segmental analysis of longitudinal strain at the basal, mid-ventricular and apical portion of the RV free ventricular wall was performed. Circumferential and radial strain parameters of both RV and LV were determined in short axis view at a basal, a mid-ventricular and an apical section of the ventricle (Figure 1B). To ensure a standardized analysis for each subject, the basal slice in short axis view was defined as the first slice below the atrioventricular level showing a circumferential myocardial enclosing, the mid-ventricular slice was localized at the level of both papillary muscles and the apical slice at an equidistant location between the basal and mid-ventricular level apical to the mid-ventricular slice.

For FT analysis, endocardial contours were drawn manually in end-diastolic images by one skilled observer with subsequent software-driven automatic tracking of the endocardial contour throughout the entire cardiac cycle. The quality of automatic tracking was checked and contours were manually adjusted and tracking repeated if necessary. Strain and strain rate were derived on a segmental and on a global level on axial and short axis images.

### Statistical analysis

For statistical analysis, the PASW 18.0 software package (IBM) was used. All continuous data are given as mean ± standard deviation. To test for significant differences between patient and control groups as well as patient subgroups, analysis of variance (ANOVA) testing was performed. For post-hoc analysis of multiple paired-group comparisons, Bonferroni correction was applied. For all patients with a diagnosis of ARVC or borderline ARVC, a subgroup analysis was performed for different ranges of ejection fractions, graded according to the cut-off values as defined in the TF criteria, and for the absence vs. the presence of wall motion abnormalities.

Patients with available data from genotyping for PKP-2 mutations were compared for differences in conventional functional and strain parameters depending on their mutation status using the Mann–Whitney-U-Test.

The diagnostic accuracy of global functional and strain parameters was evaluated for all patients with a diagnosis of ARVC (n=20) using receiver operating curve (ROC) analysis with ≥ 4 points in TF criteria (major criteria = 2 points, minor criteria = 1 point) as the gold standard. Optimal cut-off values were identified to achieve a high sensitivity and a reasonable high specificity (≥70%) for the detection of the disease. A p-value of <0.05 was regarded statistically significant.

## Results

### Basic demographic and volumetric data

Demographic characteristics and basic diagnostic data of the four study groups including the mean Task Force Score (TFS), the severity of wall motion abnormalities (WMA) and the strength of subgroups divided depending on ejection fraction subdivisions (EF) as well as RV and LV volumetric data are presented in Table 1.

**Table 1:** **Basic demographics, volumetric data and patient characteristics**

	**Healthy volunteers**	**ARVC**	**Borderline ARVC**	**pos. family history**
**n**	10	20	30	22
**Sex (m/w)**	5/5	17/3	19/11	16/6
**Age**	24.3 ± 3.0	50.7 ± 16.9^*,#^	41.4 ± 14.5^*,#^	29.3 ± 11.7
**Weight (kg)**	66.4 ± 9.4	81.3 ± 17.3	80.7 ± 16.7	80.3 ± 15.7
**Height (cm)**	176.1 ± 9.1	178.4 ± 8.0	175.6 ± 8.5	179.3 ± 9.0
**Body surface area (m** ^**2**^ **)**	1.81 ± 0.17	1.99 ± 0.22	1.96 ± 0.22	1.99 ± 0.22
**RVEDVI (ml/m** ^**2**^ **)**	88.1 ± 12.8	124.4 ± 40.4^*,$,#^	100.4 ± 31.9	93.2 ± 15.0
**RVESVI (ml/m** ^**2**^ **)**	36.7 ± 6.4	78.0 ± 39.8^**,$,##^	53.2 ± 25.0	45.7 ± 10.8
**RVEF (%)**	58.3 ± 4.8	40.4 ± 10.9^**,$$,##^	49.2 ± 8.4^*^	52.6 ± 6.9
**LVEDVI (ml/m** ^**2**^ **)**	79.0 ± 8.2	84.3 ± 18.8	89.4 ± 20.6	88.7 ± 13.2
**LVESVI (ml/m** ^**2**^ **)**	28.8 ± 4.5	33.5 ± 14.5	37.7 ± 13.3	35.4 ± 7.1
**LVEF (%)**	63.6 ± 4.2	60.7 ± 9.2	58.2 ± 7.1	61.3 ± 5.2
**Task force score**	0	5.5 ± 1.4	1.4 ± 0.8	1.5 ± 0.7
**WMA (n)**	10/0/0/0/0	7/0/1/4/8	23/0/0/4/3	20/0/0/0/2
**RVEF > 45/45 > RVEF > 40/RVEF < 40**	10/0/0	8/4/8	22/5/3	20/2/0

*Abbreviations:* *RVEDVI* Right Ventricular End-diastolic Volume Index, *RVESVI* Right Ventricular End-systolic Volume Index, *RVEF* Right Ventricular Ejection Fraction, *LVEDVI* Left Ventricular End-diastolic Volume Index, *LVESVI* Left Ventricular End-systolic Volume Index, *LVEF* Left Ventricular Ejection Fraction *WMA* Wall Motion Abnormalities.

WMA is graded in the following order: no WMA/Hypokinesia/Akinesia/Dyskinesia/ Aneurysm.

* significant difference compared to healthy volunteers.

$ significant difference compared to borderline ARVC patients.

# significant difference compared to subjects with positive family history for ARVC.

level of significance * p < 0.05; ** p < 0.01; $$ p < 0.01; ## p < 0.01.

Healthy volunteers and subjects with a positive family history of ARVC were significantly younger than patients with a diagnosis of ARVC and borderline ARVC.

**Figure 2:**
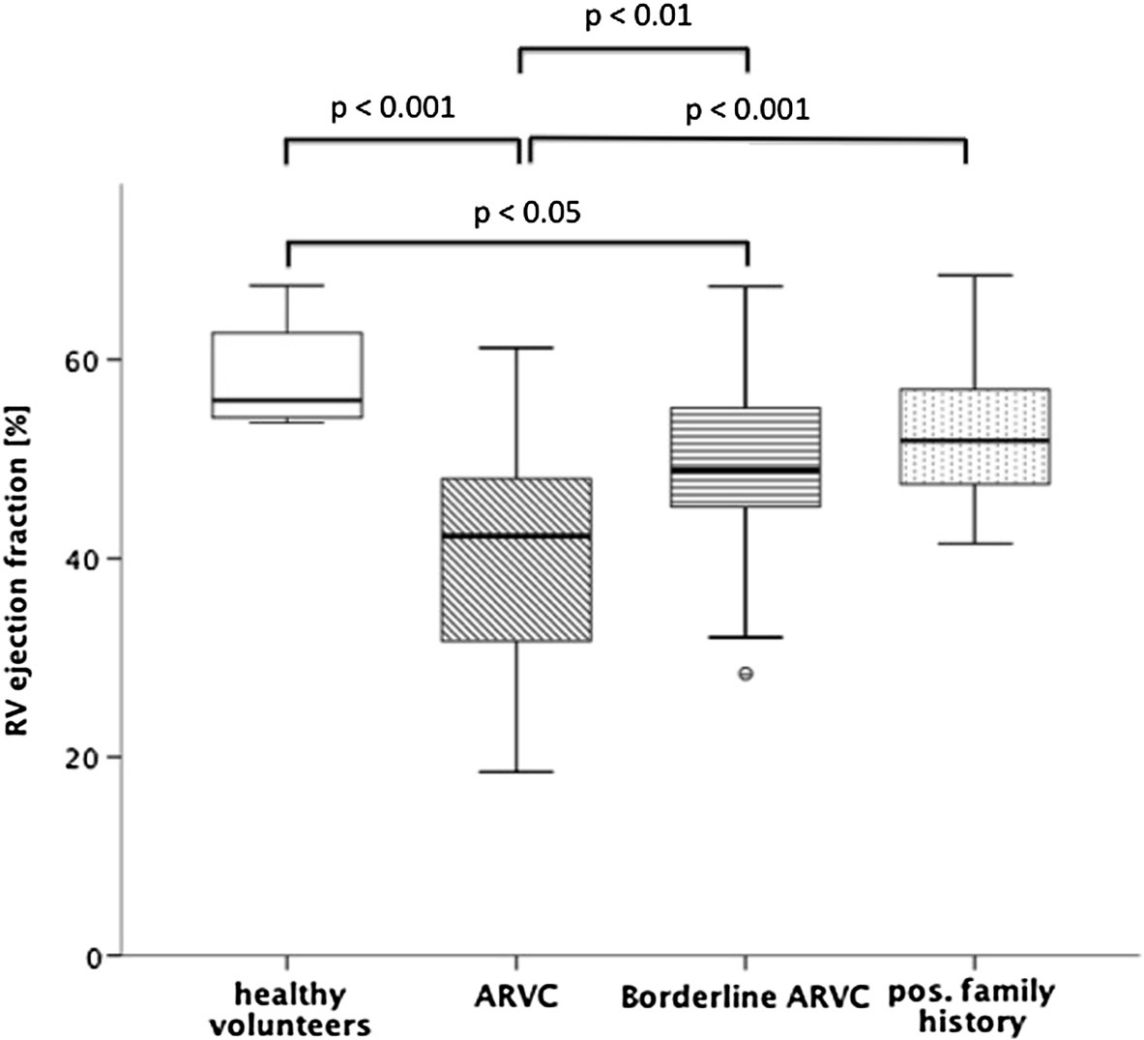
**Comparison of right ventricular ejection fraction between healthy volunteers and patient groups.**

In ARVC patients, RVEDVI and RVESVI were significantly higher and RVEF was significantly reduced compared to all other groups (Figure 2). In borderline ARVC patients, RVEF was significantly reduced compared to healthy volunteers. In contrast, LVEDVI, LVESVI and LVEF did not differ significantly among the 4 groups.

### CMR based strain analysis using feature tracking

**Figure 3:**
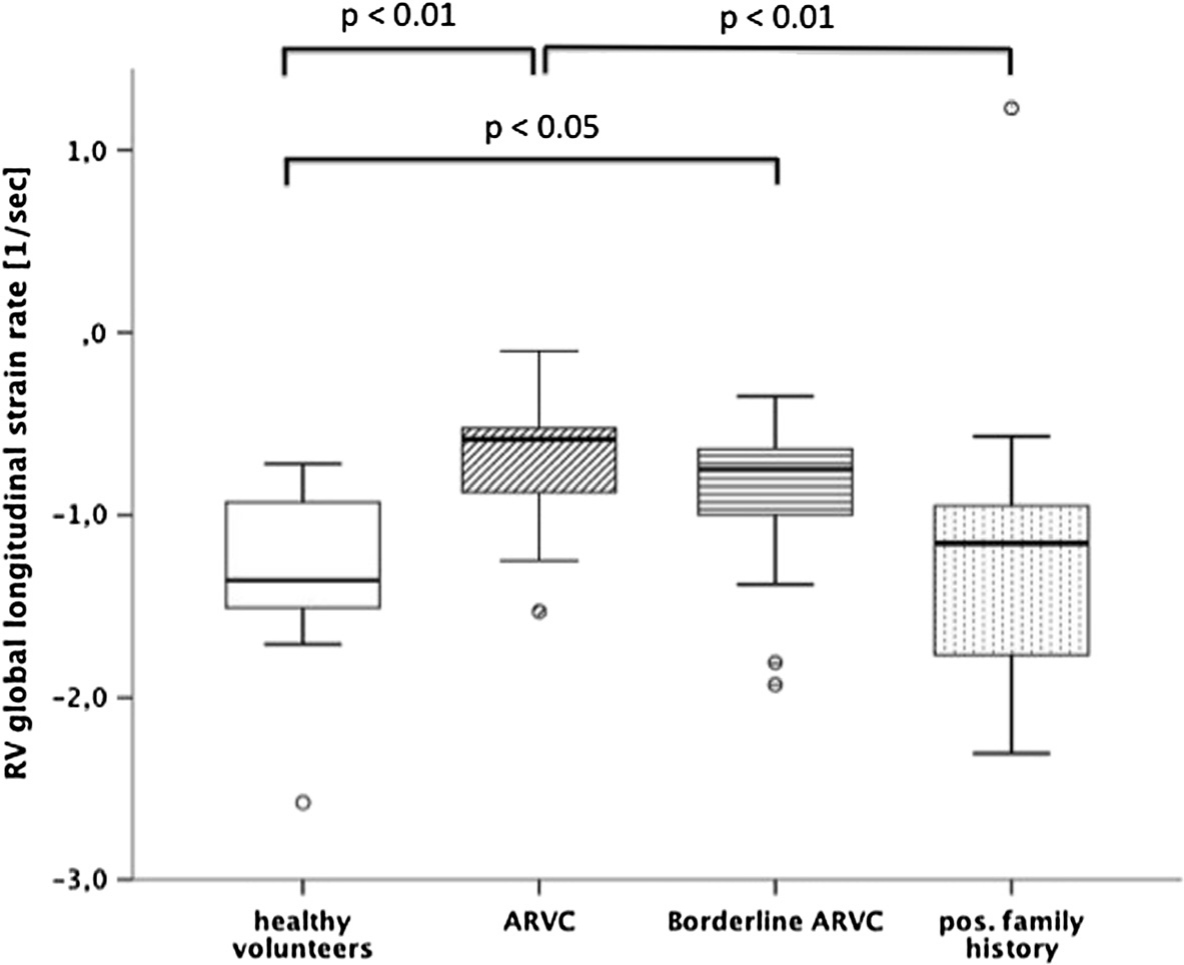
**Comparison of right ventricular global longitudinal strain rates between healthy volunteers and patient groups.**

Right ventricular global strain and strain rate parameters are given in Table 2. RV global longitudinal strain rate was significantly reduced in ARVC patients in comparison with healthy volunteers (−0.68 ± 0.36% sec^−1^ vs. -1.38 ± 0.52 sec^−1^; p ≤ 0.005) and subjects with a positive family history (−1.22 ± 0.73 sec^−1^; p ≤ 0.01) (Figure 3). Also, borderline ARVC patients showed significantly reduced longitudinal strain rates compared with healthy volunteers (−0.85 ± 0.36 sec^−1^ vs. -1.38 ± 0.52 sec^−1^, respectively; p ≤ 0.05).

**Table 2:** **Comparison of right ventricular strain and strain rate parameters between healthy volunteers, patient groups and subjects with positive family history for ARVC**

			**Healthy volunteers**	**ARVC**	**Borderline ARVC**	**pos. family history**
RV-strain	longitudinal		−19.3 ± 6	−12.7 ± 7.3^###^	−13.5 ± 6.9^###^	−20.4 ± 4.8
	circumferential	basal	−9.2 ± 3.6	−5.1 ± 2.7^*^	−6.5 ± 3.1	−7.3 ± 3.9
		medial	−8 ± 2.8	−5 ± 3.1	−6.3 ± 3.2	−7.5 ± 3.4
		apical	−12.5 ± 4.5	−11.5 ± 6.6	−11.4 ± 8.3	−10 ± 5.2
	radial	basal	16.4 ± 7.1	11.9 ± 7.6	15.3 ± 10.2	18.2 ± 9.6
		medial	12.5 ± 4.2	13.2 ± 8.4	12.5 ± 9.8	13.1 ± 6.6
		apical	12 ± 7.2	14.6 ± 14.6	19.4 ± 15.3	12.5 ± 10.5
RV-strainrate	global longitudinal		- 1.38 ± 0.52	−0.68 ± 0.36^***##^	−0.85 ± 0.36*	1.22 ± 0.73
	circumferential	basal	−0.61 ± 0.21	−0.31 ± 0.13^***,###^	−0.45 ± 0.18	−0.53 ± 0.26
		medial	- 0.49 ± 0.23	−0.31 ± 0.14^#^	−0.47 ± 0.23	−0.51 ± 0.17
		apical	−0.9 ± 0.38	−0.78 ± 0.32	−0.92 ± 0.69	−0.79 ± 0.25
	radial	basal	0.82 ± 0.32	0.46 ± 0.25^*,#^	0.73 ± 0.38	0.79 ± 0.33
		medial	0.71 ± 0.25	0,64 ± 0,26	0.67 ± 0.31	0.72 ± 0.26
		apical	0.8 ± 0.22	0.82 ± 0.46	1.1 ± 0.5	1.05 ± 0.43

* significant difference compared to healthy volunteers.

$ significant difference compared to borderline ARVC patients.

# significant difference compared to subjects with positive family history for ARVC.

level of significance * p < 0.05; ** p < 0.01; *** p < 0.005; ## p < 0.01; ### p < 0.005.

**Figure 4:**
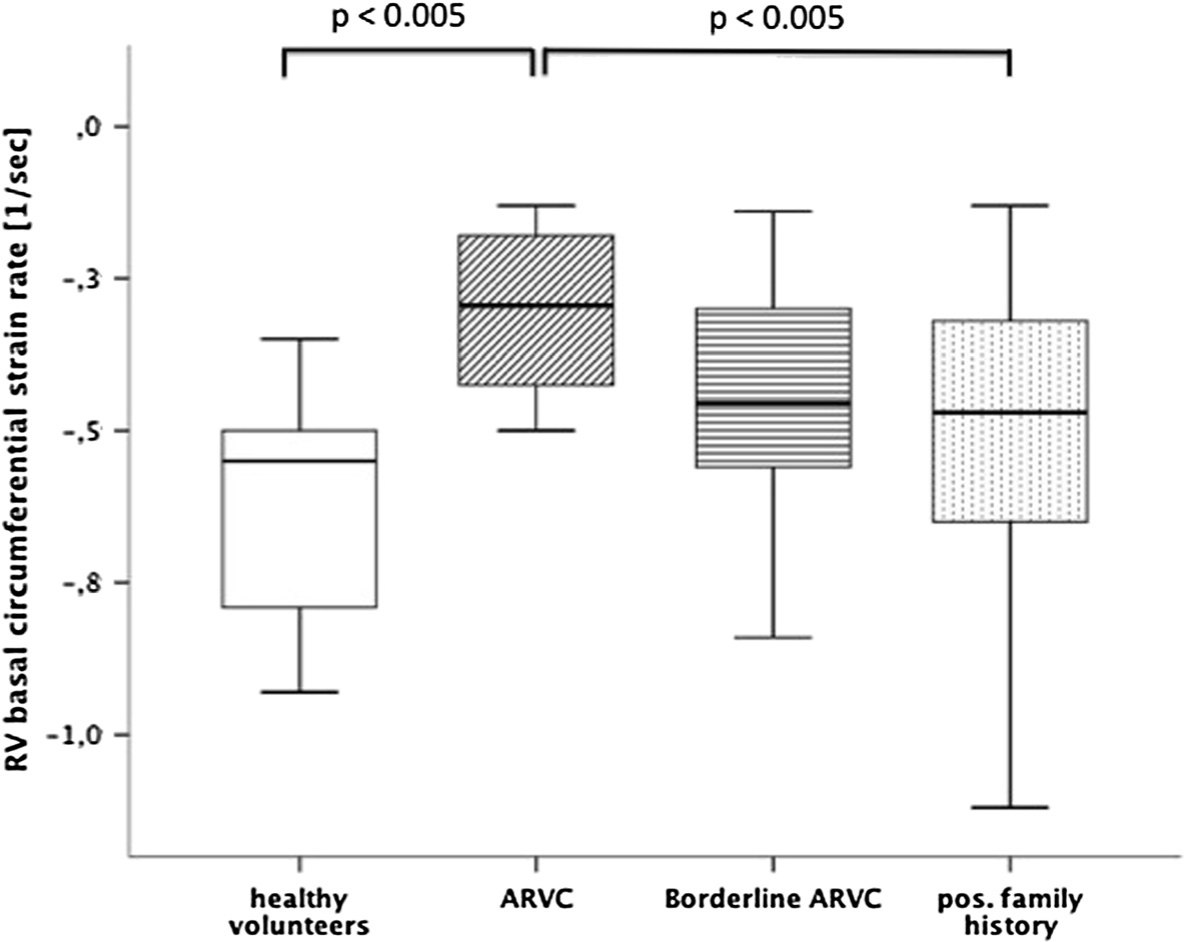
**Comparison of right ventricular basal circumferential strain rates between healthy volunteers and patient groups.**

Furthermore, in patients with a confirmed diagnosis of ARVC circumferential (Figure 4) and radial strain rates were significantly impaired at the basal level in short axis view when compared to healthy volunteers or subjects with a positive family history. The same was true for the circumferential strain values at the basal level compared to healthy volunteers.

Segmental longitudinal strain analysis of the free right ventricular wall revealed significantly lower strain rates in the basal and apical segments for patients with ARVC (basal: −1.4 ± 0.8 sec^−1^, p < 0.005; apical: −0.8 ± 0.1 sec^−1^, p < 0.05) and in the basal segment for borderline ARVC (basal: −1,8 ± 0.2 sec^−1^, p < 0.005; apical: −1.1 ± 0.1 sec^−1^) compared to healthy volunteers (basal: − 5.5 ± 2.2 sec^−1^; apical: −1.6 ± 0.2 sec^−1^). At the medial segment, no significant differences were found.

**Figure 5:**
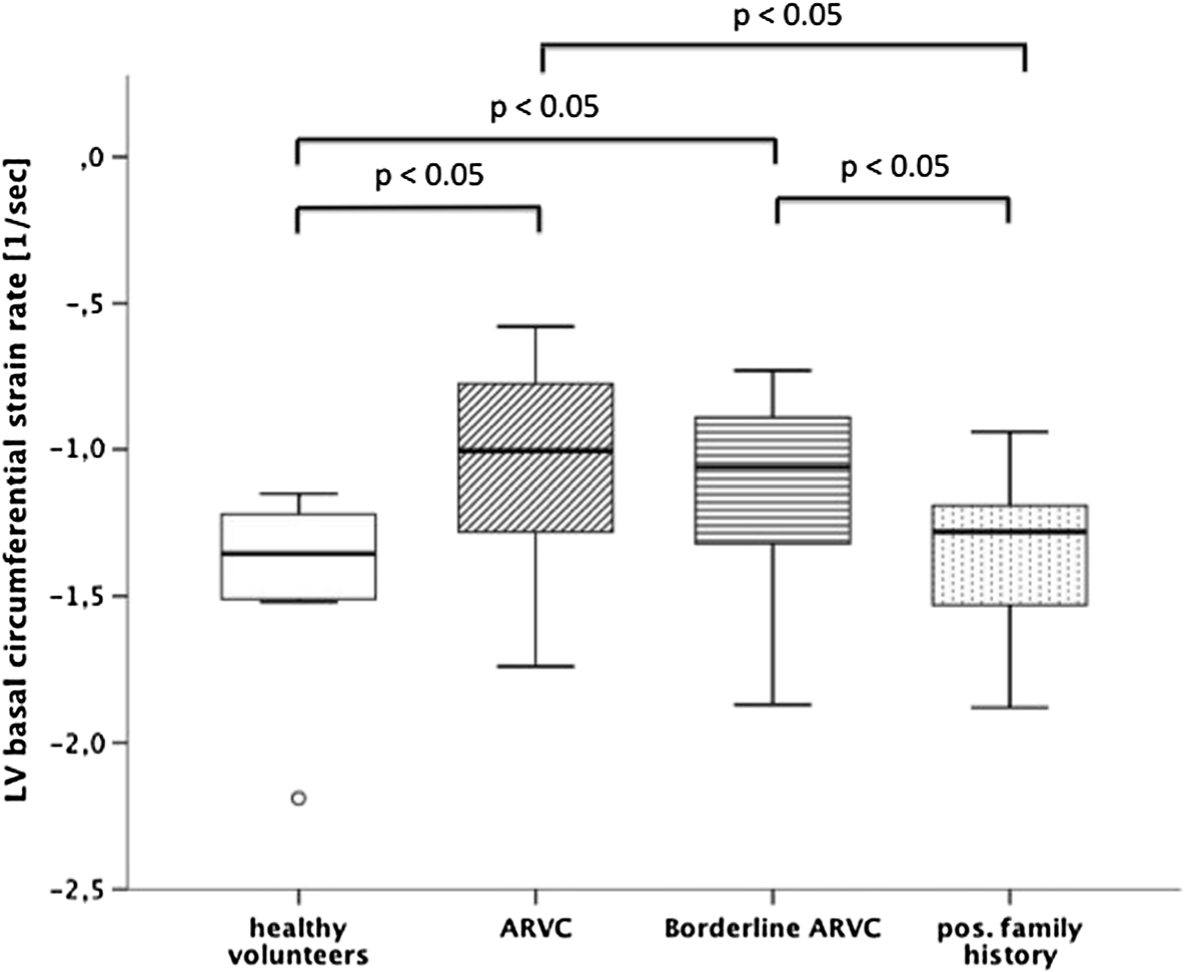
**Comparison of left ventricular basal circumferential strain rates between healthy volunteers and patient groups.**

On left ventricular strain analysis, the longitudinal strain rate (−0.7 ± 0.2 vs. -1.2 ± 0.5 sec^−1^; p < 0.05) as well as the circumferential strain rate at the basal level (−1.1 ± 0.3 vs. -1.4 ± 0.3 sec^−1^; p < 0.05) were significantly reduced in ARVC patients compared to healthy volunteers. Additionally, patients with borderline ARVC had reduced peak circumferential strain rates at the basal left ventricular level compared to healthy volunteers (−1.1 ± 0.3 vs. -1.4 ± 0.3 sec^−1^; p < 0.05, Figure 5).

All other right and global left ventricular strain and strain rate parameters were not significantly different on post-hoc analysis between patient groups and healthy volunteers when applying Bonferroni correction.

### Subgroup analysis

**Figure 6:**
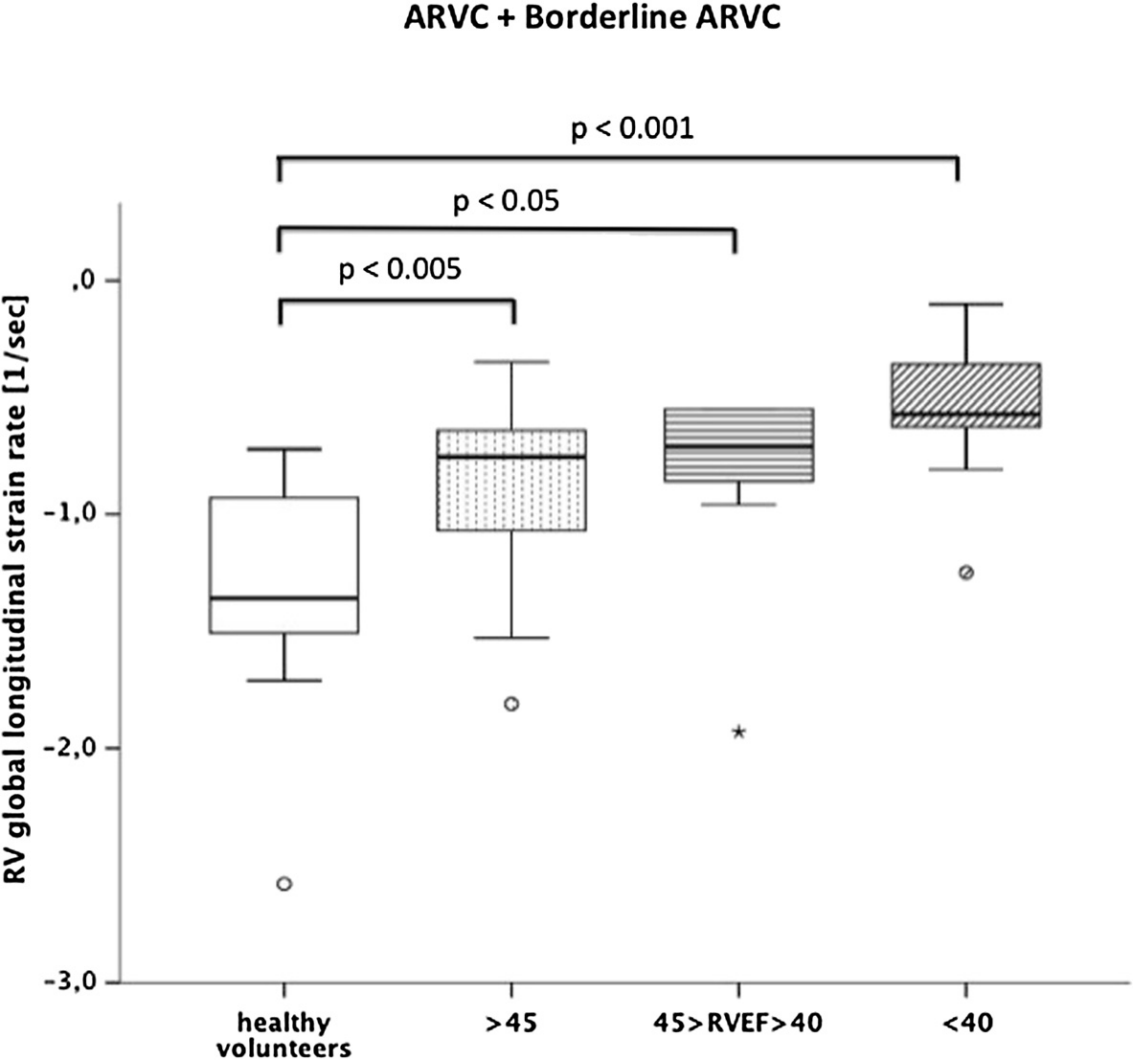
**Comparison of right ventricular global longitudinal strain rates between healthy volunteers vs. ARVC and borderline ARVC patient subgroups divided based on right ventricular ejection fraction.**

**Figure 7:**
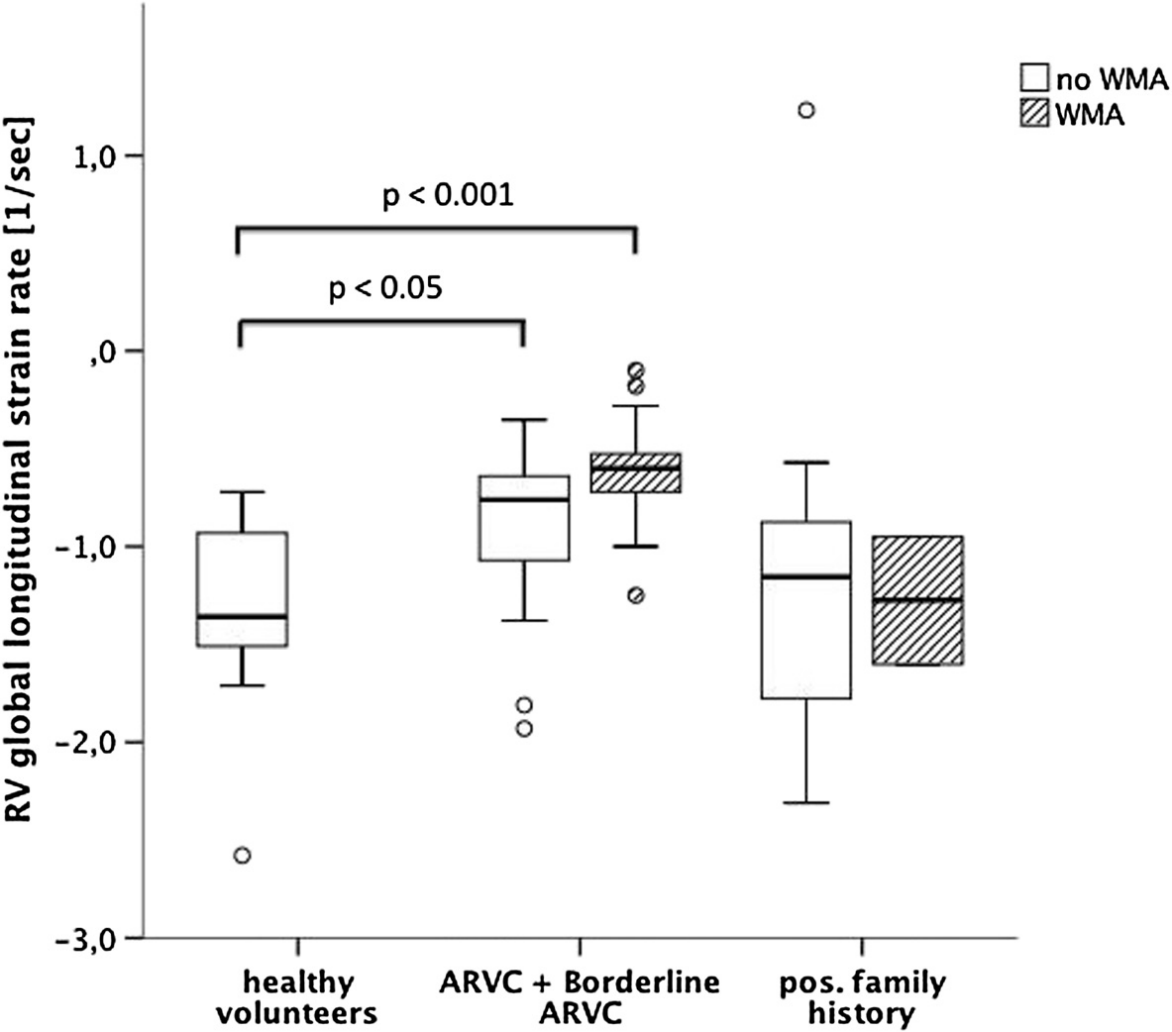
**Comparison of right ventricular global longitudinal strain rates between healthy volunteers and patient subgroups divided based on the visual detection of wall motion abnormalities.**

In patients with ARVC or borderline ARVC, RV global longitudinal strain rates were reduced most significantly in patients with an ejection fraction below 40% (n=11; −0.5 ± 0.3 sec^−1^; p < 0.001; Figure 6). However, even patients with a preserved ejection fraction of more than 45% (n=30) proved to have a significant reduction in RV global longitudinal strain rates when compared to healthy volunteers (−0.9 ± 0.3 vs. -1.4 ± 0.5 sec^−1^; p < 0.005). Similarly, even in patients with no noticeable wall motion abnormalities (n=30), global longitudinal RV strain rates proved to be significantly impaired when compared to healthy volunteers (−0.9 ± 0.4 vs. -1.4 ± 0.5 sec^−1^; p < 0.05; Figure 7). In patients with evident wall motion abnormalities (n=20) the mean longitudinal strain rate was reduced to −0.6 ± 0.3 sec^−1^ (p < 0.0001 for the comparison with healthy volunteers). No such differences could be confirmed for the other assessed strain parameters.

**Figure 8:**
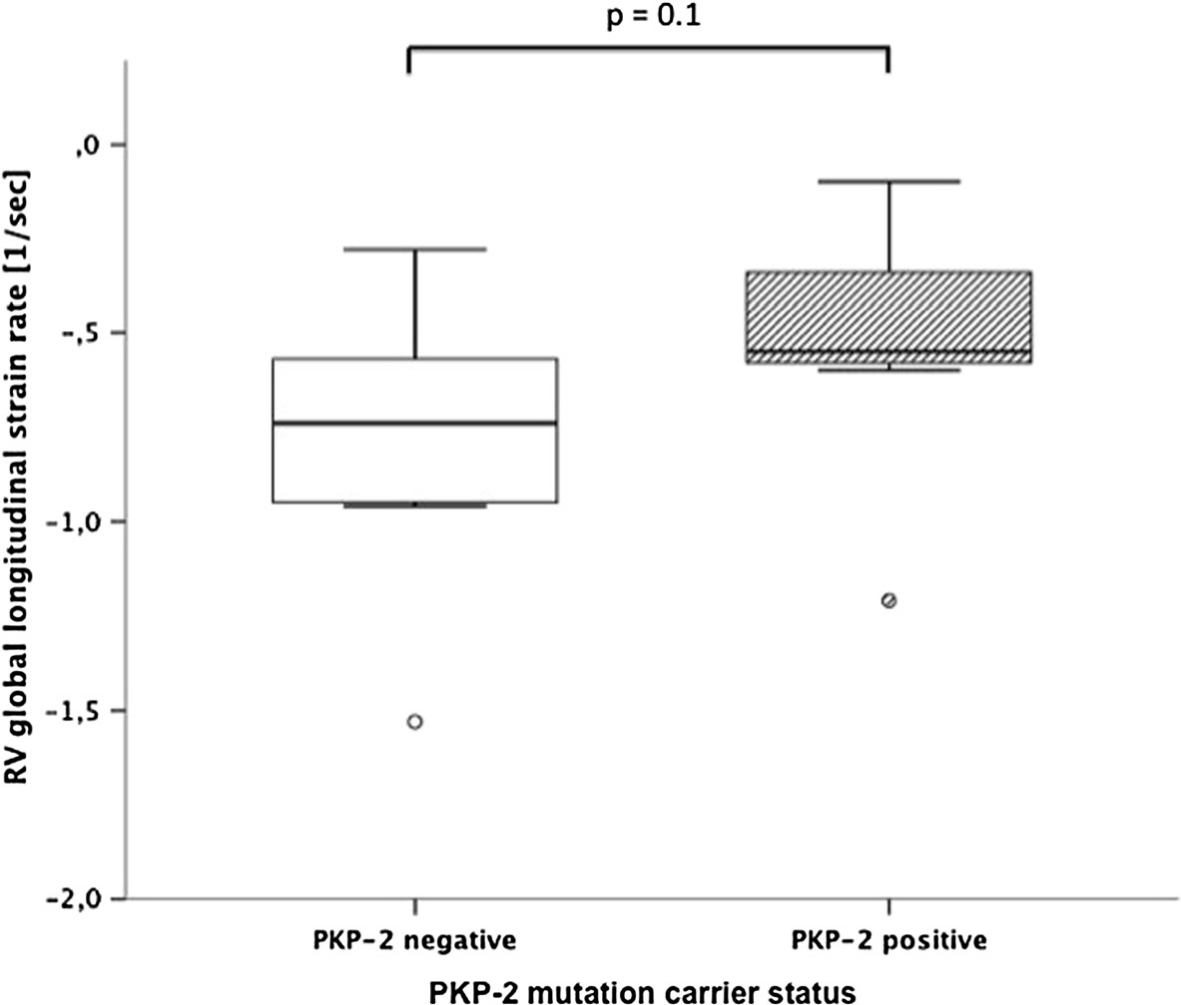
**Comparison of right ventricular global longitudinal strain rates between ARVC patients subdivided based on PKP-2 mutation carrier status.**

In patients, in which genotyping for the presence of PKP-2 mutations had been performed, a clear trend towards a more severe reduction in RV global longitudinal strain rate in mutation carriers (n=7) could be observed (−0.5 ± 0.4 vs. -0.8 ± 0.4 sec^−1^), although the observed difference did not reach the level of significance (Figure 8).

### ROC analysis

**Figure 9:**
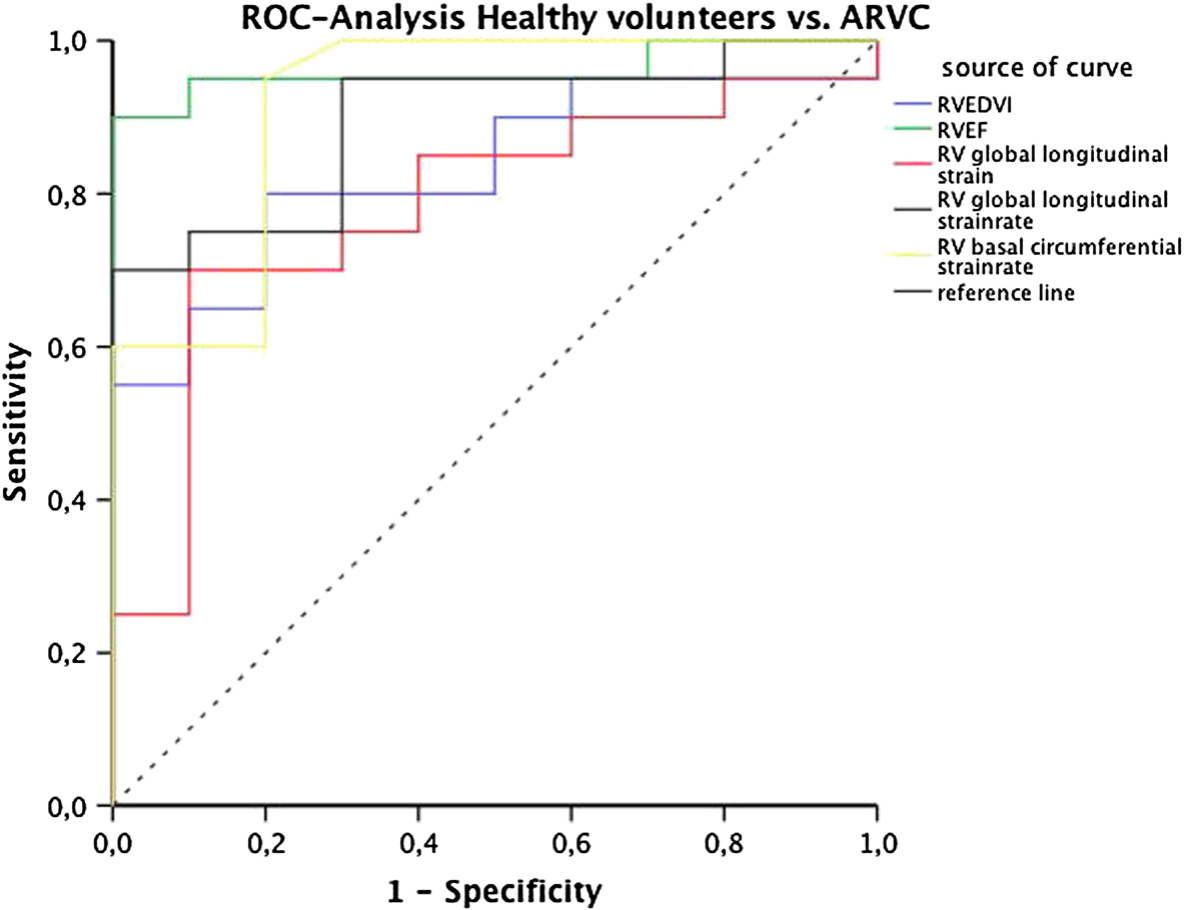
**Receiver operating curves of selected right ventricular conventional functional and strain parameters for differentiating healthy volunteers from ARVC patient.**

On ROC analysis (Table 3, Figure 9) RV ejection fraction was the strongest discriminator between ARVC patients and healthy volunteers. However, also the RV global longitudinal strain rate, the segmental longitudinal strain rate in the basal segment and the RV circumferential strain rate at the basal level proved to be very good discriminators between ARVC patients and healthy volunteers (AUC: 0.9, 0.92 and 0.92, respectively) outperforming RVEDVI with an AUC of 0.83. The AUC for the lowest longitudinal segmental strain rate of the free RV wall was 0.91. Table 4 lists the corresponding cut-off values for the parameters with high AUCs and the resulting sensitivity and specificity for the detection of ARVC. Cut-off values were chosen to achieve high sensitivity values at reasonable high (≥70%) specificity values.

**Table 3:** **Receiver operating curve characteristics (AUC – area under the curve, 95% CL – Confidence Levels) for conventional right ventricular functional parameters, right ventricular strain and strain rate parameters**

			**AUC**	**Significance**	**Lower 95% CL**	**Upper 95% CL**
RVEF (%)			0.96*	0.0001	0.89	1.0
RVEDVI			0.83*	0.004	0.68	0.98
RV-strain	global longitudinal		0.78*	0.014	0.60	0.96
	circumferential	basal	0.82*	0.005	0.65	1.0
		medial	0.79*	0.012	0.61	0.96
		apical	0.52	0.86	0.31	0.73
	radial	basal	0.68	0.113	0.49	0.87
		medial	0.5	1.0	0.29	0.70
		apical	0.48	0.86	0.26	0.70
RV-strainrate	global longitudinal		0.9*	0.001	0.78	1.0
	circumferential	basal	0.92*	0.0005	0.81	1.0
		medial	0.78*	0.015	0.60	0.95
		apical	0.52	0.84	0.30	0.74
	radial	basal	0.82*	0.005	0.67	0.98
		medial	0.59	0.42	0.37	0.81
		apical	0.56	0.6	0.35	0.77

* p < 0.05.

**Table 4:** **Optimal cut-off values of selected right ventricular conventional functional and strain parameters and corresponding levels of sensitivity and specificity**

**Parameter**	**Cut-off**	**Sensitivity (%)**	**Specificity (%)**
RVEDVI (ml/m^2^)	98.5	80	80
RVEF (%)	54	95	90
Global RV longitudinal strain rate (%/sec)	−1.28	95	70
RV longitudinal strain rate at basal segment (%/sec)	−2.41	95	70
Lowest RV segmental longitudinal strain rate (%/sec)	−0.99	90	70
RV basal circumferential strain rate (%/sec)	−0.49	95	80

## Discussion

A number of echocardiographic studies have demonstrated the feasibility and have advocated the use of strain analysis of the right ventricle in the diagnostic work-up of patients with a suspected diagnosis of ARVC [26-28]. While CMR forms an integral part in the modern diagnostic work-up of ARVC for the quantification of conventional functional parameters, CMR-based strain analysis, though feasible, is still technically demanding and not routinely performed [17,29]. With the advent of strain analysis based on conventional cine CMR using feature-tracking, strain analysis has now become far more accessible [18].

To our knowledge, this study is the first to assess the value of CMR feature-tracking-derived strain analysis in the diagnostic workup of patients suspected of suffering from arrhythmogenic right ventricular cardiomyopathy. We were able to demonstrate, that FT-derived global longitudinal as well as circumferential strain and strain rates at the basal level of the right ventricle were significantly reduced in patients with a confirmed diagnosis of ARVC compared to healthy volunteers and family relatives. Even if not as pronounced, the global longitudinal strain rate also proved to be significantly reduced in borderline ARVC patients compared to healthy volunteers.

Moreover, we found the global longitudinal strain rate to be reduced in ARVC and borderline ARVC patients, in which the RV ejection fraction was still preserved. This is in accordance with a number of echocardiographic studies, which have acclaimed the usefulness of strain rate imaging in the evaluation of subclinical myocardial disease in various cardiac pathologies [10-15]. In general, strain rate describes the rate of change in myocardial deformation with respect to time and has been found to reflect myocardial contractility best, while strain parameters are pre-load and after-load dependent and may change with ventricular dimensions [9]. Its high sensitivity for subtle deteriorations of myocardial function makes strain rate a promising parameter in the detection of early disease stages, when global functional parameters may still be normal. In a recent study Teske et al. demonstrated that echocardiographic strain analysis detects abnormal deformation in a considerable number of asymptomatic ARVC gene carriers compared to healthy controls [26].

Another recognized advantage of strain analysis is the fact that it allows for obtaining objective, quantifiable measures that are less operator-dependent than a mere visual wall motion analysis. Visual wall motion analysis is prone to significant inter-observer variability, particularly in case of the right ventricle due to its complex contraction pattern [30,31]. In our study, global longitudinal strain rate was not only significantly reduced in ARVC and borderline ARVC patients with overt wall motion abnormalities, but also patients with no apparent wall motion abnormalities proved to have significantly reduced global RV longitudinal strain rate.

At a segmental level, we found a significant reduction in strain rate values at the basal and the apical segment of the right ventricular wall. In conjunction with the right ventricular outflow tract these two segments form the so-called triangle of dysplasia and represent the sites of predilection for the manifestation of ARVC-related functional and structural changes [1]. However, while echocardiographic strain studies are usually performed at a segmental level, segmental strain values derived from CMR-based feature tracking need to be interpreted with caution. A number of FT-derived strain studies have shown that the reproducibility of segmental strain measures is only low [32,33]. Also, our segmental strain data are in conflict with previous echocardiographic data, which found the segmental strain to be significantly higher in the apical segment compared to the basal segment [34]. In our study, strain and strain rates were highest in the basal segment.

In contrast to the limited reproducibility of segmental strain measures, various FT-derived strain studies have demonstrated good inter-study and inter-observer reproducibility for global longitudinal and particularly circumferential strain measures [20,32]. Accordingly, global RV longitudinal strain obtained in our healthy volunteers was in good agreement to measures published in literature [32].

On intra-observer analysis global circumferential strain measures have been reported to be equally well reproducible, whereas results for global RV longitudinal strain measures are somewhat conflicting with coefficients of variation ranging from 9.7% [20] to 28.7% [33]. While no echocardiographic strain analysis had been performed in our study, previous studies have found good agreement between FT-derived global longitudinal strain values and data derived from speckle tracking [19,20].

Data on the validity of FT-derived strain rate measures, however, are still somewhat scarce. In a recent study, Orwat et al. found the measures of RV strain rate to be less reproducible than global strain parameters, insinuating that this might be due to the relatively limited temporal resolution of cine CMR data [19]. So far, no data exists about the extent to which strain rate measures are influenced by the temporal resolution of cine CMR data, which is considerably lower than the frame rates used in echocardiographic strain analyses.

In a subgroup of our ARVC-patients, data from genotyping for the presence of plakophilin-2 mutations was available. The Plakophilin-2 gene encodes for a structural component of desmosomes, which form junctions between myocardial cells [1]. Mutations in the Plakophilin-2 have been found to account for a considerable number of inherited ARVC cases [35]. In our study, there was a trend towards a more pronounced reduction in global RV longitudinal strain-rate in mutation carriers. Presence of PKP-2 mutations in ARVC correlates with earlier onset of symptoms and arrhythmias [36]. However, the phenotypic expression of PKP-2 mutations is known to vary considerably [1].

On left ventricular strain analysis, global longitudinal strain rate and circumferential strain rate at the basal level proved to be significantly impaired in our patients with ARVC. This finding is in line with previous studies that have found left ventricular involvement to be a common feature in ARVC patients even at an early disease stage [28].

By definition, all ARVC patients in our study fulfilled the criteria established by the task force group. As right ventricular dilatation and impaired ejection fraction are part of the disease defining criteria, these features were present in the majority of our patients. For that reason the AUC values for RVEF and RVEDVI were naturally high on ROC analysis and RVEF turned out to be the overall best discriminator between healthy volunteers and patients with ARVC. However, also global RV longitudinal strain rate, the lowest segmental strain rate of the free right ventricular wall and the circumferential strain rate at the base of the RV proved to be strong discriminators with high AUC values. Of all strain parameters the circumferential strain rate at the base of the RV had the highest diagnostic accuracy with a cut-off value at −0.49%/sec.

In echocardiographic studies, the lowest segmental strain of the free right ventricular wall has been reported to be the best quantitative parameter for discrimination between healthy controls and patients with a confirmed diagnosis of ARVC [27].

### Limitations

Even though our results are very promising, they still need to be interpreted with caution. With the above-mentioned limited evidence for the validity of FT-derived strain rate parameters, further studies are needed to evaluate the effect of varying CMR cine temporal resolution on strain rate. Improvements to the feature-tracking algorithm are needed to improve reproducibility of right ventricular and particularly segmental strain analysis [33]. As no echocardiographic strain analysis had been performed in our subjects, a direct comparison of both methods with respect to their diagnostic value is still missing. Thus, no conclusive statement can be made on the superiority of the diagnostic accuracy of either imaging modality. While Teske et al. reported slightly higher values of AUC for global and segmental RV strain values, Aneq et al. reported that longitudinal strain measurements using speckle tracking may be less reliable in more advanced disease stages [27,28].

Our results are based on a relatively small number of ARVC patients and healthy volunteers. Among our patients, we did not control for the time of disease onset or definite diagnosis in relation to the timing of the CMR exam, thus disease duration may have varied between groups. In a number of our patients the indication for CMR was not for disease detection but for monitoring the progression of an already known ARVC. For that reason, some of our patients presented with a rather advanced disease stage and the overall age of our population was higher than in other cohorts that have been reported on in literature [5,26,27].

The control and patients groups were not age-matched. Instead, the control group was significantly younger compared to both patient groups. This may have introduced a bias and led to an overestimation of the ARVC-related impairment of longitudinal strain parameters in patients, as longitudinal myocardial shortening has been reported to decrease with age, even though the decrease is only moderate [37]. Conversely, circumferential function has been reported to increase with age [38]. Thus any differences of this parameter found in our study population may even have been slightly underestimated.

Our results were not controlled for the treatment with antiarrhythmic drugs. While Teske et al. found no significant difference in echocardiographic strain parameters between patients with and without drug therapy other functional and volumetric parameters, such as heart rate, iso-volumetric acceleration and RV inflow tract size have been described to be influenced by antiarrhythmic drug therapy [27].

## Conclusion

In our study, we were able to demonstrate that CMR based strain analysis using feature tracking may serve as a valuable tool to detect and quantify impaired myocardial function in ARVC patients beyond conventional functional parameters. FT-derived strain measures allowed for the differentiation between manifest or borderline ARVC on the one hand and healthy volunteers or family relatives on the other hand. Even patients, in whom ejection fraction was preserved or no wall motion abnormalities had been detected on visual analysis, were found to have impaired strain measures. In view of these findings FT-derived CMR strain analysis promises to become a powerful measure to further objectify diagnosis and detect ARVC.

In order to confirm and establish its potential role in the detection of early disease stages and to assess potential prognostic implications of FT-derived strain parameters in ARVC patients, further studies with a prospective and longitudinal design are warranted. Also, the role of strain imaging in differentiating ARVC from other common differential diagnoses like RVOT tachycardia or Brugada syndrome needs to be further clarified [39].
